# Early experience of seven hepatocellular carcinoma cases treated with regorafenib

**DOI:** 10.1002/ccr3.1791

**Published:** 2018-10-11

**Authors:** Shinsuke Uchikawa, Tomokazu Kawaoka, Hiroshi Aikata, Kenichiro Kodama, Yuki Inagaki, Masahiro Hatooka, Kei Morio, Takashi Nakahara, Eisuke Murakami, Akira Hiramatsu, Masataka Tsuge, Michio Imamura, Yoshiiku Kawakami, Kazuaki Chayama

**Affiliations:** ^1^ Department of Gastroenterology and Metabolism, Applied Life Sciences Institute of Biomedical & Health Sciences Hiroshima University Hiroshima Japan

**Keywords:** hepatocellular carcinoma, regorafenib

## Abstract

Regorafenib became second‐line treatment for the patients with sorafenib refractory. In our study, two patients could not continue regorafenib for its adverse effects. It was suggested that appropriate use criteria of regorafenib should be observed and manage adverse effects earlier.

## 1 INTRODUCTION

There were no systemic treatments for patients with hepatocellular carcinoma (HCC) whose disease progress during sorafenib treatment. The RESORCE study proved that regorafenib provides survival benefit in HCC patients progressing on sorafenib treatment as second‐line chemotherapy. We assessed the efficacy and safety of regorafenib in seven patients with advanced HCC.

Hepatocellular carcinoma is one of the leading causes of cancer‐related death worldwide.^1^ HCC commonly occurs in patients with chronic hepatitis or liver cirrhosis because of hepatitis B or C virus, alcohol, nonalcoholic steatohepatitis, or diabetes.^2^ The treatment of HCC follows well‐established guidelines.^3^, ^4^, ^5^ For patients who are not or who are no longer candidates of locoregional therapy, the oral multikinase inhibitor sorafenib was the systemic treatment available for the treatment of advanced HCC.

Regorafenib is molecular‐targeted drug that inhibits angiogenic kinase and stromal RTKs VEGFR1, VEGFR2 and VEGFR3, TIE2, and PDGFR‐beta that promote tumor neovascularization, vessel stabilization, and lymphatic vessel formation and play an important role in the tumor microenvironment.^6^ The RESORCE study proved that regorafenib provides survival benefit in HCC patients progressing on sorafenib treatment as second‐line chemotherapy.^7^ Regorafenib has been approved for clinical use since June 2017 as a second‐line treatment for patients with advanced HCC and progression on sorafenib.

In this case report, we reported the efficacy and safety of regorafenib after 1‐cycle treatment.

## 2 CASE

### 2.1 Patients

A total of seven patients with advanced HCC progressed after sorafenib treatment at Hiroshima University were received regorafenib. They must have Child‐Pugh A liver function and ECOG PS 0‐1 and sorafenib tolerance (They must have tolerated sorafenib (≥400 mg daily for at least 20 of the 28 days before discontinuation).

### 2.2 Regorafenib treatment

Patients received 160 mg regorafenib orally once daily for 3 weeks in each 4‐week cycle. Treatment interruptions and dose reductions were permitted for adverse drug reactions.

### 2.3 Evaluation of response to regorafenib

The radiological response was evaluated by computed tomography every month after regorafenib initiation with the Response Evaluation Criteria In Solid Tumors (RECIST) and modified RECIST (mRECIST). Adverse drug reactions were defined according to the Common Terminology Criteria for Adverse Events version 4.0 (CTCAE 4.0).

### 2.4 Patient background characteristics

The clinical characteristics of seven patients at the start of sorafenib treatment are summarized in Table 1. All seven patients were male. One patient had stage II HCC, one patient had stage III HCC, and five patients had stage IVB HCC. The median of alpha‐fetoprotein (AFP) before sorafenib treatment was 17 034 ng/mL, and the median of PIVKA‐II before sorafenib treatment was 2275 mAU/mL. No patients had macroscopic vascular invasion (MVI). The median of duration of sorafenib was 3.4 months, the daily dose of sorafenib was 530.6 mg, and the last dose of sorafenib was 800 mg daily in three patients and 400 mg daily in four patients. The clinical characteristics of six patients at the start of regorafenib are summarized in Table 2. Two patients had stage III HCC, five patients had stage IVB HCC. No patients had MVI. The median of AFP before regorafenib treatment were 27 550 ng/mL and the median of PIVKA‐II before regorafenib treatment were 33 988 mAU/mL. The starting regorafenib dose was 160 mg daily in all seven patients.

**Table 1:** Background of seven patients treated with regorafenib at the start of sorafenib

Case	Age	Gender	Etiology	Child‐Pugh score	PS	HCC stage	MVI	Extrahepatic metastasis	Tumor marker (before sorafenib)		Initial dose of sorafenib	Daily dose before discontinuation of sorafenib	Average daily dose of sorafenib	Duration of sorafenib treatment	Adverse effects of sorafenib
									AFP (ng/mL)	PIVKA‐2 (mAU/mL)					
1	83	M	NBNC	5 (A)	0	II	None	None	30.8	6943	800	400	585	1.4	None
2	65	M	HCV	5 (A)	0	IVb	None	Lung	17 034	2275	800	400	531	3.4	None
								Peritoneum							
3	71	M	NBNC	6 (A)	1	IVb	None	Lung	161.1	44 588	800	400	540	2	Anorexia
								Peritoneum							Hypertension
4	60	M	HCV	5 (A)	0	III	None	None	22 830	93	800	800	488	5.7	Hypertension
5	68	M	NBNC	5 (A)	1	IVb	None	Lung	19 920	1069	800	800	469	27.7	None
								Bone							
6	79	M	NBNC	5 (A)	0	IVb	None	Lung	19	301	800	400	445	7.6	Hypertension
7	67	M	NBNC	6 (A)	0	IVb	None	Lung	1 647 200	2717	800	800	800	2.5	Rush
								Bone							

AFP, alpha fetoprotein; F, female; HBV, hepatitis B virus; HCC, hepatocellular carcinoma; HCV, hepatitis C virus; M, male; MVI, macroscopic vascular invasion; NBNC, non‐hepatitis B virus and non‐hepatitis C virus; PIVKA‐2; protein induced by vitamin K absence or antagonist‐2.

**Table 2:** Background and evaluation of regorafenib after 1‐cycle treatment

Case	Child‐Pugh score	PS	HCC stage	MVI	Extrahepatic metastasis	Initial dose of regorafenib	AFP (ng/mL)		PIVKA‐2 (mAU/mL)		Radiological evaluation after 1‐cycle treatment		Adverse events after 1‐cycle treatment (grade 3/4)	Continuation of regorafenib
							Before	After	Before	After	RECIST	mRECIST		
1	6 (A)	0	III	None	None	160	5.5	2.8	13 108	483	PR	PR	None	Continuation without dose reduction
2	6 (A)	0	IVb	None	Lung	160	39 978	21 520	33 988	29 613	SD	SD	Hypophosphatemia	Continuation without dose reduction
					Peritoneum								Hypertension	
3	6 (A)	1	IVb	None	Lung	160	1433.1	5310	92 580	305 300	SD	SD	HFSR	Discontinuation after 7 d
					Peritoneum								Elevated AST	
4	6 (A)	0	III	None	None	160	36 373.6	31 550	3492	18 158	SD	SD	None	Continuation without dose reduction
5	5 (A)	1	IVb	None	Lung	160	27 550	143 970	153 450	135 630	PD	PD	None	Continuation without dose reduction
					Bone									
6	5 (A)	0	IVb	None	Lung	160	168.2	51.1	17 453	38 342	PD	PD	None	Continuation without dose reduction
7	6 (A)	1	IVb	None	Lung	160	7 444 000	1 655 000	102 540	87 400	PD	PD	None	Continuation without dose reduction
					Bone									

AFP, alpha fetoprotein; F, female; HBV, hepatitis B virus; HCC, hepatocellular carcinoma; HCV, hepatitis C virus; M, male; mRECIST, modified RECIST; MVI, macroscopic vascular invasion; NBNC, non–hepatitis B virus and non–hepatitis C virus; PIVKA‐2; protein induced by vitamin K absence or antagonist; PR, partial response; RECIST, response evaluation criteria in solid tumors.

### 2.5 Response to regorafenib treatment

Based on RECIST and mRECIST, the proportion of patients with a complete response (CR), partial response (PR), stable disease (SD), and progressive disease (PD) was 0% (n = 0), 14.3% (n = 1), 42.9% (n = 3), and 42.9% (n = 3) at the initial evaluation after 1‐cycle treatment. The overall response rate was 14.3%, and the disease control rate was 57.1%.

### 2.6 Safety and tolerability of regorafenib

Six patients could receive regorafenib without discontinuation and dose reduction during a 1‐cycle therapy. One patient discontinued because of severe adverse effect and deterioration of liver function and performance status. During the 1‐cycle regorafenib treatment, six patients had any adverse events. The adverse events in 1 cycle, occurring at any grade, were hand‐foot skin reaction (n = 4; 57.1%), hypertension (n = 3; 42.3%), elevated AST (n = 4; 57.1%), hypophosphatemia (n = 3; 42.3%), diarrhea (n = 3; 42.3%), elevated lipase (n = 3; 42.3%), low platelet (n = 3; 42.3%), anorexia (n = 2; 28.6%), elevated total bilirubin (n = 2; 28.6%), elevated ALP (n = 2; 28.6%), renal failure (n = 1; 14.3%), fever (n = 1; 14.3%), elevated ALT (n = 1; 14.3%), elevated amylase (n = 1; 14.3), and vomiting (n = 1; 14.3%). CTCAE grade 3 adverse event were in two cases. 1case had CTCAE grade 3 elevated AST and hand‐foot skin reaction. In this case, general condition was deteriorated and regorafenib treatment was discontinued in 7 days. The other case had CTCAE grade 3 hypertension and hypophosphatemia. In this case, the patients could continue regorafenib treatment because of antihypertensive drug and oral phosphate supplement.

### 2.7 The case of discontinuation of regorafenib because of grade 3 liver disfunction

**Figure 1:**
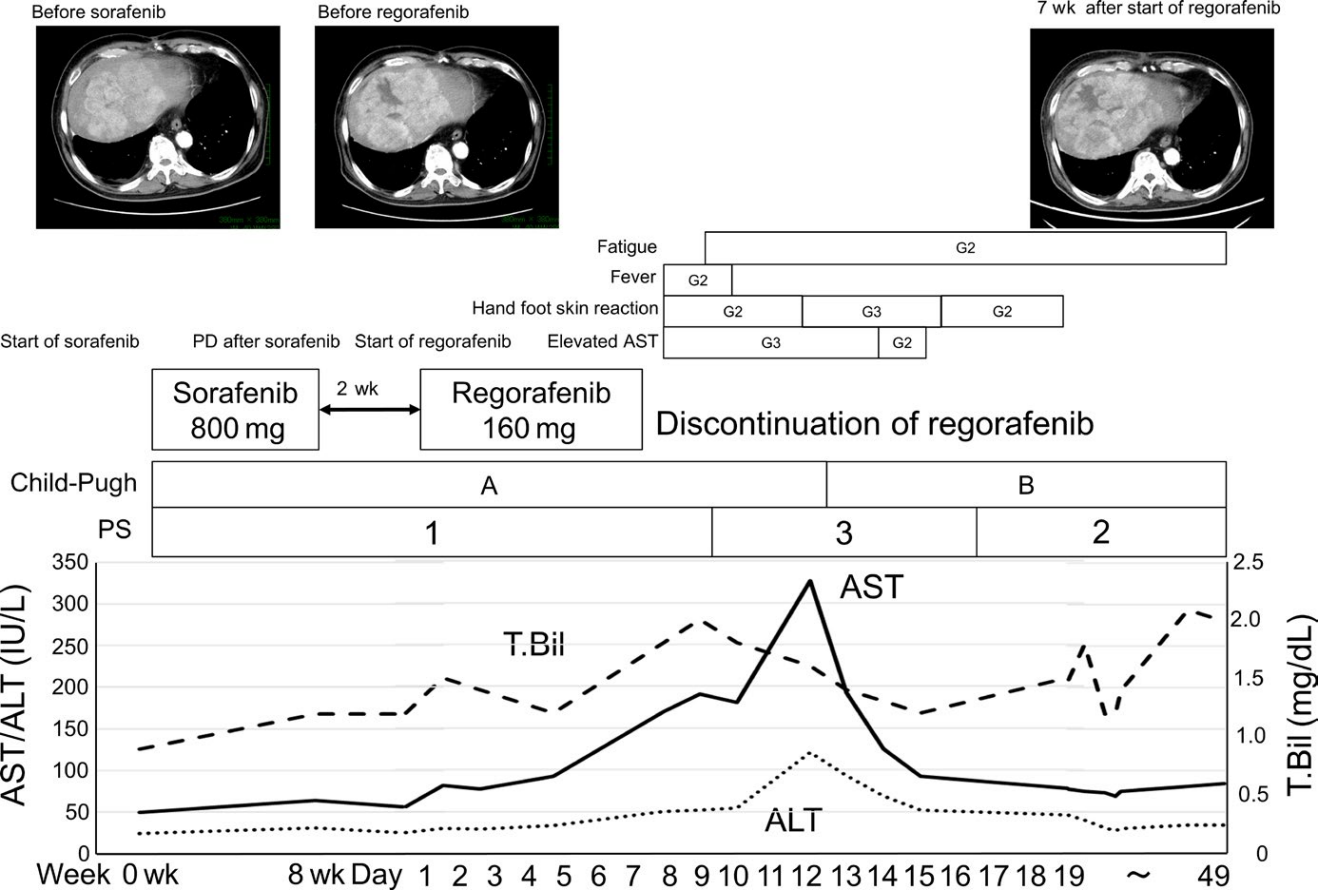
The case of discontinuation of regorafenib because of CTCAE grade 3 elevated liver enzyme (case 3). CTCAE, Common Terminology Criteria for Adverse Events

The case was a 71‐year‐old man (Figure 1). The duration of sorafenib treatment was 2 months, the daily dose was 540 mg and the adverse effects of sorafenib were hand‐foot skin reaction (grade 2), hypertension (grade 3), and anorexia (grade 3); however, he did not have liver dysfunction as adverse effect of sorafenib. HCC progressed after sorafenib treatment. He had lung metastasis, peritoneal metastasis, and no MVI. At the progression, he had Child‐Pugh A liver function, PS 1, and tolerance of sorafenib. We assessed whether he was eligible for regorafenib, and he received an initial dose of regorafenib of 160 mg. AST showed a tendency to increase at day 2. AST elevated to 170 IU/L (grade 3) at day 7 and hand‐foot skin reaction (grade 2) appeared, and we interrupted regorafenib based on the guide for appropriate use of regorafenib. However, AST was 326 IU/L at day 12 and hand‐foot skin reaction got worse to grade 3. After then, AST decreased to 78 U/L at day 19 and hand‐foot skin reaction was cured to grade 1. Total bilirubin increased only to 1.7 mg/dL, however, we assessed that he could not continue regorafenib because of ascites, deteriorated liver function, and continuation of fatigue. In our hospital, the case of discontinuation of regorafenib was only in this case out of six cases. In this case, we interrupted regorafenib as soon as possible because of grade 3 elevated AST, but after discontinuation AST was increasing and it takes 12 days hand‐foot skin reaction and liver dysfunction to be cured.

### 2.8 The case of PR after 1‐cycle regorafenib treatment

**Figure 2:**
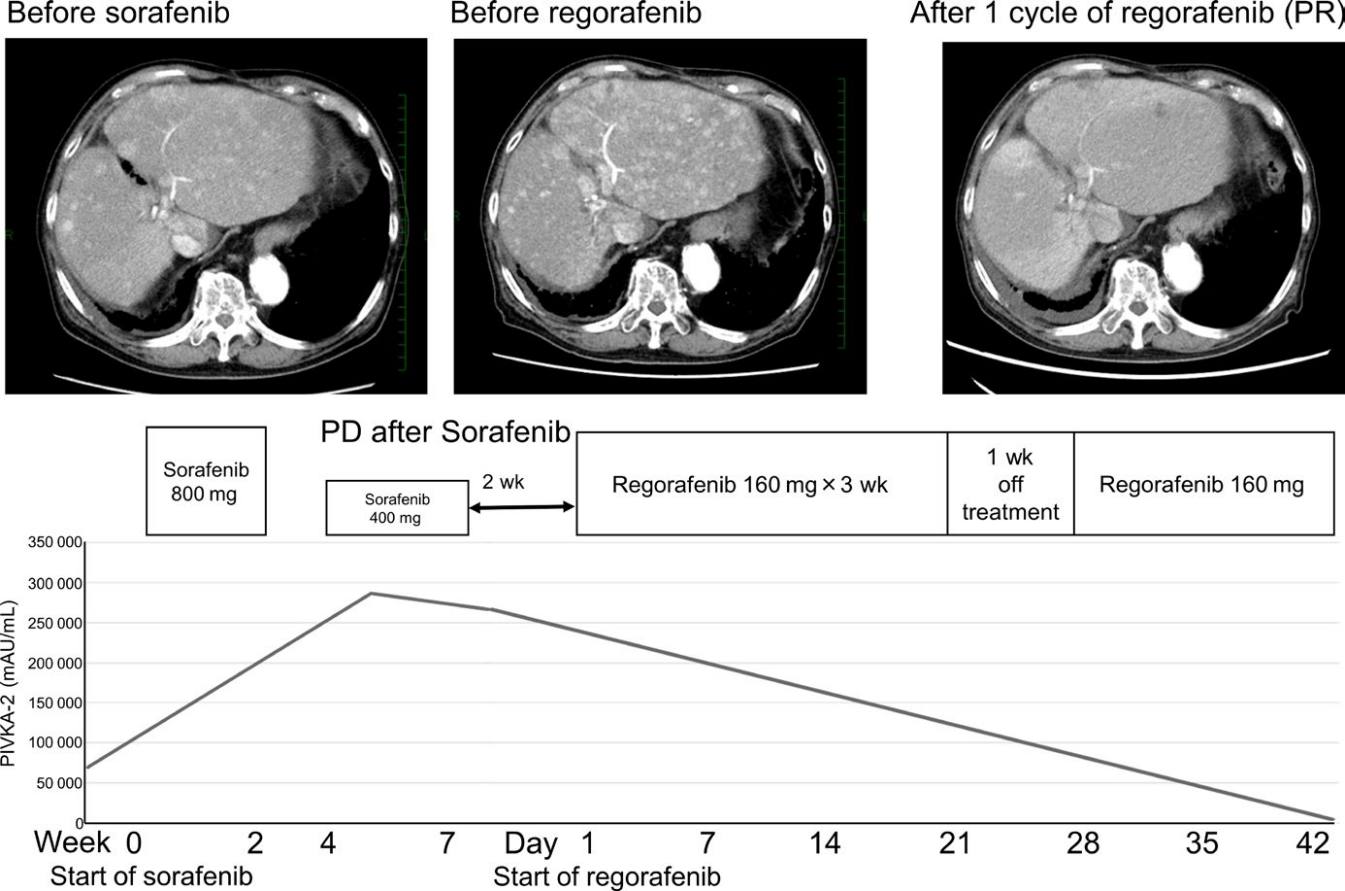
The case of PR after 1‐cycle regorafenib treatment (case 1). PR, partial response

The case was 83 years old man (Figure 2). HCC was diffused in liver without MVI and metastasis (TNM stage III). HCC progressed after sorafenib treatment for 2 months. He received initial dose of regorafenib of 160 mg. He had no adverse effects and he could continue 1‐cycle regorafenib treatment without dose reduction and discontinuation of regorafenib. After 1‐cycle regorafenib treatment, almost tumor stain disappeared, radiological evaluation was PR. PIVKA‐2 decreased from 13 108 to 483 mAU/mL.

## 3 DISCUSSION

I reported early experience of seven cases of regorafenib. All of them were eligible for regorafenib and received initial dose of regorafenib of 160 mg. In 1‐cycle evaluation, it was necessary for one patient to interrupt regorafenib because of grade 3 elevated AST. In this case, adverse effects of regorafenib were getting worse after treatment interruption. In phase I study of regorafenib, after single dosing, the median time to maximum concentration of regorafenib and its metabolite M2 was 4 hours, and that of metabolite M5 was 24 hours. Similar terminal half‐lives (*t*_1/2_) were observed for regorafenib and M2, being approximately 27 and 25 hours, respectively, and a longer *t*_1/2_ of approximately 61 hours was observed for M5.^8^ The time for metabolism of regorafenib is longer than that of sorafenib. We think that the adverse effects of regorafenib were getting worse after treatment interruption because of the longer *t*_1/2_.We should manage adverse effects sooner particularly liver dysfunction than sorafenib. In our hospital, we start regorafenib in hospitalization and we examined liver function for a week until 2 cycles.

According to Ueshima et al,^9^ sorafenib toxicities tended to reappear with successive regorafenib treatment. In our study, severe adverse effects during the sorafenib treatment did not appear; however, they appeared during the regorafenib treatment. We should be careful of the adverse effects not only which appeared during the sorafenib treatment but also which did not appear during the sorafenib treatment. In the RESORCE study, 68% patients in the regorafenib group needed interruptions or dose reduction because of adverse effects.^7^ In our study, six of the seven patients could continue regorafenib treatment without dose reduction and interruption of regorafenib in 1 cycle. However, our study was the only 1‐cycle evaluation, as the monitoring period gets longer, interruption and dose reduction may increase.

## 4 CONCLUSION

Radiological evaluation after 1‐cycle regorafenib treatment was PR in one case. In the RESORCE study, response rate was 11%. In our study response rate was 14.3%, this result was similar to the RESORCE study. It was suggested that appropriate use criteria of regorafenib should be observed and manage adverse effects earlier, particularly liver dysfunction, on regorafenib treatment.

## CONFLICT OF INTEREST

None declared.

## AUTHOR CONTRIBUTIONS

SU: drafted the manuscript. HA: approved the manuscript. TK: approved the manuscript.
